# Systematic evaluation of differential splicing tools for RNA-seq studies

**DOI:** 10.1093/bib/bbz126

**Published:** 2019-12-05

**Authors:** Arfa Mehmood, Asta Laiho, Mikko S Venäläinen, Aidan J McGlinchey, Ning Wang, Laura L Elo

**Affiliations:** 1 Turku Bioscience Centre, University of Turku and Åbo Akademi University, Turku, Finland; 2 Department of Physiology, University of Turku, Turku, Finland; 3 School of Medical Sciences, Örebro University, Örebro, Sweden

**Keywords:** RNA-seq, differential splicing, splicing events, isoform-based methods, exon-based methods, event-based methods

## Abstract

Differential splicing (DS) is a post-transcriptional biological process with critical, wide-ranging effects on a plethora of cellular activities and disease processes. To date, a number of computational approaches have been developed to identify and quantify differentially spliced genes from RNA-seq data, but a comprehensive intercomparison and appraisal of these approaches is currently lacking. In this study, we systematically evaluated 10 DS analysis tools for consistency and reproducibility, precision, recall and false discovery rate, agreement upon reported differentially spliced genes and functional enrichment. The tools were selected to represent the three different methodological categories: exon-based (DEXSeq, edgeR, JunctionSeq, limma), isoform-based (cuffdiff2, DiffSplice) and event-based methods (dSpliceType, MAJIQ, rMATS, SUPPA). Overall, all the exon-based methods and two event-based methods (MAJIQ and rMATS) scored well on the selected measures. Of the 10 tools tested, the exon-based methods performed generally better than the isoform-based and event-based methods. However, overall, the different data analysis tools performed strikingly differently across different data sets or numbers of samples.

## Background

Differential splicing (DS) enables production of various messenger RNAs (mRNAs), and thereby various protein products, from one gene [1–3]. This process allows for great complexity and diversity of mRNA and protein products without a concomitant increase in genome size. For instance, more than 90–95% of multi-exonic genes in human have been found to undergo DS [2, 4]. Dysfunction of DS has been associated with cellular dysfunction and the pathology of different diseases, especially cancer [5]. Further, DS events have been proposed as both biomarkers and potential targets for drug discovery [6].

Alternative splicing (AS) events are currently divided into five main types: skipped exons (SE), alternative 5′ (donor) splice sites (A5SS), alternative 3′ (acceptor) splice sites (A3SS), retained introns (RI) and mutually exclusive exon usage (MXE) [7]. In exon skipping, an exon is spliced out of the transcript together with its flanking introns. Exon skipping is the most prevalent AS event in higher eukaryotes, accomplishing around 40% of all AS, but is rarely encountered in lower eukaryotes [8, 9]. Alternative 3′ and 5′ splice sites selection accounts for 18% and 8% of all AS in higher eukaryotes, respectively, and it occurs when two or more splice sites are recognized at one end of an exon. Intron retention, where an intron remains in the mature mRNA transcript, is commonly observed in plants, fungi and metazoa, while in higher eukaryotes it only constitutes for around 5% of known AS events [10]. Several other less frequent, complex AS events are also recognized, the most prevalent of these being MXE, where only one of the dependent exons is being retained at a time.

The technology of RNA-sequencing (RNA-seq) has enabled the detailed analysis of the transcriptome [2] and its changes under different conditions or in different tissues. Despite its enormous utility, RNA-seq does not naturally lend itself to elucidating DS events due to the short nature of the sequencing reads (usually around 100–150 bp or shorter) [11], resulting in their possible alignment to different transcripts of the same gene [12]. However, a number of computational methods have, to date, been developed for DS analysis [4, 13–15].

Two major strategies are currently applied for DS analysis: isoform-based (used by tools such as cuffdiff2 [16] and DiffSplice [11]) or count-based [17], the latter further divided into exon-based (e.g., DEXSeq [18], edgeR [19], JunctionSeq [20] and limma [21]) or event-based methods (e.g. dSpliceType [22], MAJIQ [23], rMATS [24] and SUPPA [25]/SUPPA2 [26]) (Supplementary Figure S1). Isoform-based methods aim at reconstructing and quantifying full-length transcripts, prior to differential expression analysis. With count-based methods, the genes are usually configured into a single representation consisting of counting units that can be, for example, full or truncated exonic regions or junction regions. Counts are recorded as the number of sequencing reads falling on each counting unit and differential expression analysis is then carried out to call differentially expressed counting units. While most modern methods are able to analyse DS between different sample groups, some of the earlier tools such as MISO [27], ALEXA-Seq [28], rSeqDiff [29] and SpliceSeq [30] perform the analysis between two individual samples only, limiting their utility for many studies.

A nascent area, there has been rapid development of DS data analysis methods; however, there are still calls for the systematic evaluation of their performance [12]. Some initial comparisons have been made by the developers of tools such as JunctionSeq [20], dSpliceType [22] and rSeqDiff [29]. Additionally, a comparison of DS tools on simulated and a real RNA-seq data set on plants has been performed [17], however, unlike in vertebrates, in plants intron retention is considerably more common than exon skipping [31], leaving open the applicability of the results to higher eukaryotes.

To address the need for a comprehensive and independent assessment of DS analysis methods in higher eukaryotes, we performed a comparative analysis of 10 tools developed for the detection of DS between different conditions using several real RNA-seq data sets, from human and mouse. The first four of the tools: Cufflinks/cuffdiff2 [16, 32], DEXSeq [20], DiffSplice [11] and rMATS [24], were chosen for being already commonly applied in DS research. edgeR [19] and limma [21] were included as they are currently widely used for differential gene expression analysis and also include functionality to perform DS analysis based on exon-level read counting. Finally, four recently developed promising tools were included: dSpliceType [22], JunctionSeq [20], MAJIQ [23] and SUPPA [25] /SUPPA2 [26].

All 10 tools included in our comparison were tested using four RNA-seq data sets. The first two: human prostate cancer (PCa) data set (*n* = 28) [33] and human hepatocellular carcinoma (HCa) data set (*n* = 100) [34] were chosen for the reasonable number of samples to enable investigation of the effect of the number of samples on the results. The other two data sets included several qPCR-validated splicing events. First of these, the mouse validated set (MVS), compared wild-type mice to those with knockouts of epithelial splicing regulatory proteins (Esrps) and included 28 genes with qPCR-validated exon-skipping events [35]. The second validated set, the human-validated data set (HVS), compared two human prostate cancer cell lines and included 32 qPCR-validated DS genes [24].

### Overview of DS methods

Table 1 summarizes the 10 tools used in the comparison, representing the isoform-based, exon-based and event-based approaches. A short description of each tool is given below; for a more detailed presentation, the reader is referred to the original publications.

**Table 1:** Overview of the DS analysis methods used in the evaluation

Method	Version	Programming language used	Reference sequence used	Approach	Annotation	Experimental designs supported	Reference
Cufflinks/cuffdiff2	2.2.1	C++	Genome	isoform-based	Yes and *de novo*	Two groups	[16]
DiffSplice	0.1.2beta	C++	No	isoform-based	*Ab initio*	Two groups + blocking (1 factor)	[11]
DEXSeq	1.16.10	R/Bioconductor	Genome	exon-based	Yes	Complex designs	[18]
edgeR	3.12.1	R/Bioconductor	Genome	exon-based	Yes	Complex designs	[19]
JunctionSeq	1.3.4	R/Bioconductor	Genome	exon-based	Yes and *de novo*	Complex designs	[20]
limma	3.26.9	R/Bioconductor	Genome	exon-based	Yes	Complex designs	[21]
dSpliceType	2.0.0	Java	Genome	event-based	Yes	Two groups	[22]
MAJIQ	2.0	Python	Genome	event-based	Yes and *de novo*	Two groups	[23]
rMATS	3.2.2.beta/3.2.5	Python	Genome	event-based	Yes	Two groups, paired samples	[24]
SUPPA	2.0.0	Python	Transcriptome	event-based	Yes	Two groups, paired samples	[25]
SUPPA2	2.2.1	Python	Transcriptome	event-based	Yes	Two groups, paired samples	[26]

### Isoform-based methods

Isoform-based methods first seek to reconstruct full-length transcripts and estimate their relative abundances in each sample based on the sequencing reads. Statistical testing is then applied to identify significant differences in the relative transcript abundances between the different experimental conditions. The performance of this approach depends on accurate transcript quantification.

#### Cufflinks/cuffdiff2

Cufflinks is a pipeline consisting of different programs including cufflinks itself [32], cuffmerge and cuffdiff2 [19]. Cufflinks first performs transcript assembly by generating overlap graphs with fragments as nodes and edges connecting the compatible fragments. Transcript abundances are then estimated by maximizing the likelihood score among all possible sets of relative abundances of each isoform. Following this, cuffmerge is used for merging the assemblies across the samples to create a consensus reference. Cuffdiff2 is finally applied to detect differentially expressed genes and differential isoform usage along with promoter-preference changes between experimental conditions. The method takes into consideration the variability between the replicates and uncertainty in abundance estimation due to ambiguously mapped reads using a beta negative binomial model of fragment counts.

#### DiffSplice

DiffSplice takes a graph-based *ab initio* approach; it first reconstructs the transcriptome based on the aligned reads, then quantifies the abundance of alternative paths through the graph and finally identifies the alternative splicing modules (ASMs) [11]. ASMs are defined as those genomic regions where alternative transcripts diverge and have at least two possible paths. Abundance of the ASMs is compared between the experimental conditions using a non-parametric permutation test. DiffSplice also reports the splicing event type associated with each differential ASM.

### Count-based methods

Count-based methods include both exon-based and event-based approaches. In exon-based methods, read counts are assigned to different features, such as exons or junctions. The limitation of this approach is that it does not infer the type of the splicing event occurring in a gene but only identifies the differentially expressed exons/junctions between experimental conditions. In event-based methods, splicing events themselves are quantified by calculating the percentage spliced in (PSI) values for each event, which measure the fraction of mRNAs expressed from a gene that contains a specific form of that event [25].

#### DEXSeq

Exon-based method DEXSeq is an R/Bioconductor package developed to detect DS from RNA-seq data. The method uses a generalized linear model to model the differential usage of exons in different sample groups [20]. It assumes that the read counts in the exons follow a negative binomial distribution and controls for false discovery rate (FDR) by estimating the biological variability for each exon.

#### edgeR

edgeR is an R/Bioconductor package that can be used to analyse differential expression at the gene, exon or transcript level [19]. The exon count data is first fitted using a negative binomial generalized log-linear model, after which the differential exon usage is tested by comparing the log-fold-change of an exon to the log-fold-change of the entire gene.

#### JunctionSeq

JunctionSeq is an R/Bioconductor package, which utilizes a similar statistical strategy as DEXSeq. It enables estimation of differential exon usage as well as known or novel exon junctions [20].

#### limma

limma is an R/Bioconductor package that is widely used for differential gene expression analysis and has been extended to perform DS using exon-level count data [21]. It fits a linear model to the exon-level expression data and then tests for differential exon usage between different biological conditions. Finally, the exon-level statistics are converted to gene-level test statistics to identify DS genes.

#### dSpliceType

dSpliceType is an event-based method designed to find DS by utilizing base-wise read coverage signal data [22]. It extracts the candidate splicing events for five different event types (SE, RI, MXE, A3SS and A5SS) using the available gene annotations and the supported junction reads. For each event, it calculates the read coverage signal for each base in each replicate and normalized logarithmic ratios of the PSI between the sample groups. The method then uses a change point analysis on the PSI followed by a parametric statistical test using Schwarz Information Criterion (SIC) [36] for detecting the DS events.

#### MAJIQ

MAJIQ (Modeling Alternative Junction Inclusion Quantification) uses local splicing variations (LSVs) to quantify RNA splicing in genes. LSVs are splits in a splice graph where several edges come to or from a single exon called a reference exon [23]. The LSVs can consist of simple splicing events as well as complex transcript variations. MAJIQ uses read rate modelling, Bayesian PSI modelling and bootstrapping to report posterior relative changes in PSI values for each quantified LSV.

#### rMATS

rMATS [24] is an event-based method, which is an improved version of the original MATS method [37]. rMATS simultaneously accounts for sampling uncertainty within individuals and variability between samples by using a hierarchical framework to model the PSI of each event. The method uses a likelihood ratio test to examine whether the between-group differences of mean PSI exceed a given, user-defined threshold. We have used rMATS versions 3.2.2 and 3.2.5 in this study, the latter (rMATSTurbo) described to provide a 100-fold increase in running time compared to the older versions.

#### SUPPA

SUPPA is an event-based method that uses transcript abundances to estimate the PSI values for each DS event [25]. Transcript abundances are determined using RSEM tool [38]. In addition to the five standard types of splicing events, SUPPA also considers two other event types, alternative first exon (AF) and alternative last exon (AL). Two different versions of the method: SUPPA and SUPPA2 [26] were included in this study.

## Materials and methods

### Data sets

In the present study, four different publicly available RNA-seq data sets were analysed (Table 2). The first RNA-seq data set, referred to as PCa data set, consists of 28 normal or tumor samples from prostate cancer [33] and was downloaded from Array Express (www.ebi.ac.uk/arrayexpress/) under accession number E-MAT-567 (accessed on November 2015). The second data set, referred to as HCa data set, involves 100 human normal and tumor samples from hepatocellular carcinoma metastasis [34] and was obtained from Gene Expression Omnibus (GEO) under the accession number GSE77314 (accessed on December 2016). The third data set, referred to as MVS data set, consists of four epidermis sample replicates of double knock-outs of Esrps and wild-type mice and was obtained from GEO under the accession number GSE64357 (GSM1569076-77, GSM1569083-84, accessed on July 2016). The data set contains 28 qPCR-validated DS genes for the comparison of double knock-out mice with wild-type mice [35]. The fourth data set, referred to as HVS data set, consists of six replicates from GS689 and PC3E prostate cancer cell lines and was downloaded from Sequence Read Archive (SRA) under accession number SRS354082 (accessed on July 2017). In the associated original study [24], 32 DS genes were validated using qPCR.

**Table 2:** Summary of RNA-seq data sets used in the comparison

Dataset name	Database IDs	Database	Number of samples	Library type	Read length	Organism	Reference	Number of qPCR-validated DS genes
PCa	E-MAT-567	Array Express	28	Paired	90	*Homo sapiens*	[33]	2
HCa	GSE77314	Gene Expression Omnibus	100	Paired	100	*Homo sapiens*	[34]	–
MVS	GSM1569076–77, GSM1569083–84	Gene Expression Omnibus	4	Paired	100	*Mus musculus*	[35]	28
HVS	SRS354082	Sequence Read Archive	6	Paired	101	*Homo sapiens*	[24]	32

### Genome and transcriptome

The reference sequences and annotation files for Ensembl GRCh37 (*Homo sapiens*) and NCBIM37 (*Mus musculus*) genomes were downloaded from Illumina igenomes (https://support.illumina.com/sequencing/sequencing_software/igenome.html). Sequences for transcriptome reference file of GRCh37 were downloaded in fasta format from Ensembl (GRCh37: release 83) and indexed using bowtie2 [39].

### Pre-processing of data

The sra files downloaded from SRA or GEO were converted to fastq files using the sratoolkit.2.8.0 [40] and the quality of the reads was analysed using FastQC v0.11.3 tool [41]. In the PCa data set, the low quality reads were trimmed using trimgalore v0.4.1 [42] to a length greater than 20 bp. rMATS has a restriction that it can only perform DS analysis if it is provided with reads of the same length, therefore for rMATS, the reads were additionally truncated to length of 80 bp using the script provided with rMATS. The reads were mapped to the reference genome (Ensembl *Homo sapiens*: GRCh37, *Mus musculus*: NCBIM37) with STAR v2.6.1b [43] using default settings. A summary of the total and mapped reads in each data set is provided in Supplementary Table S1.

The tools used to identify DS required different types of input files and the reference files for each tool were prepared according to the description available with the tool. Samtools v1.2 [44] was used when there was a need to convert from bam to sam alignment file format.

For the purpose of general quality overview, we produced gene-level read counts using the featureCounts tool [45] and normalized them using the Trimmed Mean of M values (TMM) method [46]. Principal component analysis based on the normalized count values was used to confirm that samples in all of the data sets clustered according to the sample groups (Supplementary Figure S2).

### Execution of the DS tools

In this study, we systematically evaluated 10 DS analysis tools. The tools were selected to represent the three different methodological categories: isoform-based (cuffdiff2, DiffSplice), exon-based (DEXSeq, edgeR, JunctionSeq, limma) and event-based methods (dSpliceType, MAJIQ, rMATS, SUPPA). The DS tools were run using the default settings. The description and the commands used to run the programs are provided in the Supplementary File.

### Evaluation of methods

In our first comparison setting, the consistency of the splicing tools was tested using the PCa and HCa human data sets. The results provided by different tools at the level of isoforms, exons or events were aggregated to gene level in order to compare the methods. Details of the aggregation approach are given in the Supplementary File.

For selecting the DS genes for each method the FDR threshold was set at 0.05. Where ranks of genes were considered, ranking was done based on FDR for most of the methods. For DiffSplice, the method’s own test statistic was used since this is the only statistic it provides whereas for cuffdiff2, the results were ranked according to *P*-value due to very few findings with FDR below 1. Where needed, test statistic was used as a secondary ranking criteria.

To calculate the precision and recall of the methods, we compared samples between tumor and normal group with varying numbers of replicates in each data set. Let DS_full_ denote the DS genes in the complete data set and let DS_subset_ denote the DS genes in the subset of the data. The precision was defined as

$$ Precision\left({DS}_{full},{DS}_{subset}\right)=\frac{\mid {DS}_{full}\cap {DS}_{subset}\mid }{\mid {DS}_{subset}\mid }. $$

Correspondingly, the recall was defined as

$$ Recall\left({DS}_{full},{DS}_{subset}\right)=\frac{\mid {DS}_{full}\cap {DS}_{subset}\mid }{\mid {DS}_{full}\mid }. $$

FDR was assessed in mock comparisons by selecting randomly without replacement from the normal group two artificial sample groups to be compared. The process was repeated 10 times for each different subset size. The findings of these mock comparisons were considered as false positives (FP). To estimate the FDR, the number of FPs detected were divided by the median number of detections in the real comparison with the same number of samples. FDR values were truncated to 1, if they were greater than 1.

In our second comparison setting, we compared the detections in the mouse data set (MVS) and in the human data set (HVS) to the 28 and 32 qPCR-validated DS genes, respectively.

For evaluating the performance of the tools at different sequencing depths, the HVS data set was downsampled to subsets of 20 to 100 million reads with increments of 20 million reads. Precision and recall were calculated as above with the genes detected by each tool in the original HVS data set considered as the complete set.

### Functional enrichment analysis

Functional enrichment analysis was carried out to detect the most enriched Gene Ontology (GO) biological processes across the different methods. The top 500 genes from each method were analysed for enrichment using the R/Bioconductor package topGO [47] using the classic method (each GO category is tested independently) and Fisher’s exact test. dSpliceType was excluded from this comparison as it allowed listing genes only until FDR of 0.05 and provided less than 500 genes in each data set. The results were summarized by collecting the *P*-values of the GO terms that were among the top 10 most enriched terms with at least one of the methods. The *P*-values were then used as input for hierarchical clustering and heatmap visualizations.

## Results

**Figure 1:**
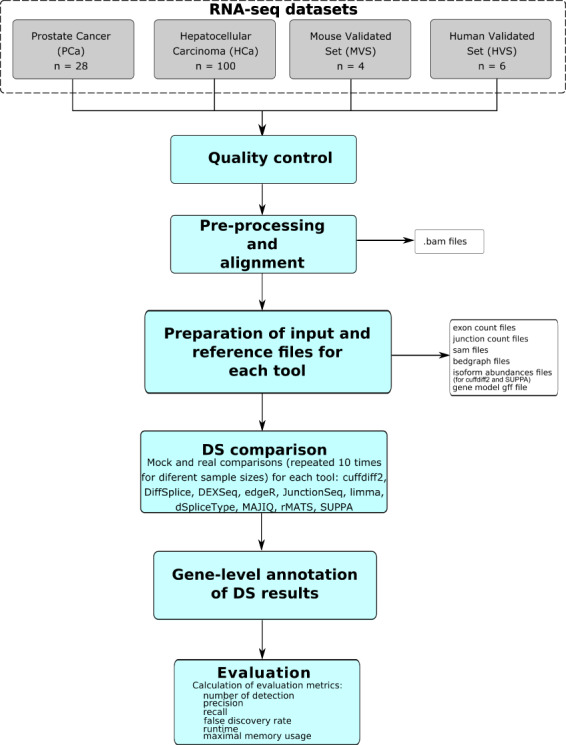
Schematic illustration of the comparison of DS tools. In total, 10 different tools were assessed in four different RNA-seq data sets.

We tested 10 different DS tools on four different RNA-seq data sets. The PCa and HCa data sets were used for the assessment of consistency and reproducibility of the tools by performing true and mock comparisons. In the true comparisons, the analyses were performed on the complete data sets as well as on smaller subsets of samples chosen at random without replacement from both sample groups. In the mock comparisons, the randomly chosen samples were all from the same (normal) experimental condition. Both the true and the mock comparisons were repeated 10 times for each subset size. In the MVS and HVS data sets, which contained qPCR-validated genes, the tools were assessed in their ability to retrieve the validated genes. The overall experimental design is illustrated in Figure **1**.

### Number and consistency of detections

**Figure 2:**
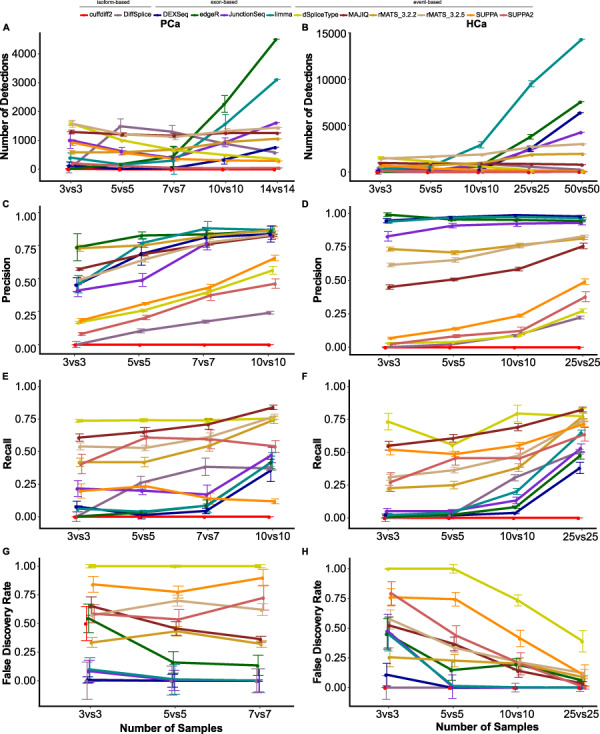
Median and standard error of the number of detections, precision, recall and FDR of the 10 compared methods in PCa and HCa data sets with different numbers of replicates. Number of DS genes in the (**A**) PCa and (**B**) HCa data set. Precision in the (**C**) PCa and (**D**) HCa data set. Recall in the (**E**) PCa and (**F**) HCa data set. FDR in the (**G**) PCa and (**H**) HCa data set. The points and error bars correspond to the median and the standard error of the 10 randomly sampled subsets for the different numbers of replicates.

Strikingly, large variability in the numbers of detections was found between the tools in the PCa and HCa data sets (Figure **2**A and B, Supplementary Figure S3A and B, Supplementary Table S2). In the complete data sets (14 samples per group in the PCa data set and 50 samples per group in the HCa data set), the number of DS genes ranged from 0 (cuffdiff2) to 4506 (edgeR) in the PCa data set and from 11 (SUPPA2) to 14 313 (limma) in the HCa data set. cuffdiff2 provided the smallest numbers of detections among the compared tools in both data sets, with most runs not reporting any findings. SUPPA/SUPPA2 also consistently produced fairly low numbers of DS genes in both data sets. Exon-based methods overall showed the highest relative variability in the number of detections between the random subsets especially at lower sample sizes. The newer version of rMATS detected more DS genes than its older version.

For most of the tools, the number of samples had a substantial effect on the number of DS genes reported; edgeR was the only tool that showed consistent increase in the number of DS genes in both data sets when the number of samples was increased. DEXSeq, JunctionSeq, limma and rMATS also followed this trend with minor deviations between consecutive subsets. DiffSplice did not show any consistent trend in the number of DS genes when the number of samples increased, while dSpliceType and SUPPA/SUPPA2 actually detected fewer DS genes with larger numbers of samples in both data sets, the newer version of the tool reporting significantly fewer DS genes than the older version. The newer version also showed increased relative SD. MAJIQ reported almost the same number of DS genes with all sample sizes.

Next, we assessed the precision and recall of the different tools by comparing the DS genes reported among the random subsets to those reported in the complete PCa and HCa data sets. Recall for a given subset was defined as the proportion of DS genes in the complete data set that were also detected in the subset (see Methods). Correspondingly, precision was defined as the proportion of DS genes detected in the subset that were also detected in the complete data set. In general, both precision and recall increased with the increase of the number of samples, except for cuffdiff2, which reported no DS genes in most subsets (Figure 2 C–F, Supplementary Figure S3C–F, Supplementary Tables S3 and S4). Overall, the tools showed considerable variation across the 10 random subsets, this being most pronounced in the PCa data set and its recall values. The exon-based methods DEXSeq, edgeR, JunctionSeq and limma in general had higher precision than the event-based and isoform-based methods, although the difference to MAJIQ and both versions of rMATS was not large in the PCa data set.

To investigate the FDR reported by the different tools, we performed mock comparisons in the PCa and HCa data sets by randomly sampling two groups from the normal experimental condition. Normal sample group was used here, as differences between the random subsets were expected to be minor compared to differences in the tumor group and thus DS genes reported in the derived mock comparisons can be considered to be FP. The FDR for each tool was estimated by scaling the number of FP by the median number of DS genes detected with the same number of samples in the corresponding real comparison. Generally, FDR and its variability across the 10 random subsets decreased as the number of samples increased (Figure **2**G and H, Supplementary Figure S3G and H, Supplementary Table S5), being clearly the lowest in the runs with the highest numbers of samples in the larger HCa data set (*n* = 10 or *n* = 25). However, the variability of the FDR values was noticeable for DiffSplice, edgeR, JunctionSeq, limma and SUPPA/SUPPA2 even at the largest sample size in the PCa data set. Similar to the real comparisons, isoform-based cuffdiff2 reported very few DS genes also in the mock comparisons, regardless of the number of samples. Isoform-based DiffSplice overall reported the lowest FDRs in both data sets, followed by the exon-based tools DEXSeq, JunctionSeq and limma. Out of the event-based tools MAJIQ and rMATS (both versions) performed better than SUPPA/SUPPA2 and dSpliceType, which reported similar numbers of DS genes across the runs in both the true and the mock comparisons, leading to very high FDR.

**Table 3:** Proportions of the different event types detected by the event-based tools among top 500 reported events.^1^

Method	Data set	% of ES events	% of RI events	% of ASS events	% of MXE events	% of AF events	% of AL events
MAJIQ	PCa	0.21	0.71	a3ss = 0.03 a5ss = 0.04			
	HCa	0.52	0.33	a3ss = 0.05 a5ss = 0.10			
rMATS v3.2.2	PCa	0.15	0.73	a3ss = 0.07 a5ss = 0.04	0.01		
	HCa	0.51	0.35	a3ss = 0.07 a5ss = 0.06	0.02		
rMATS v3.2.5	PCa	0.16	0.61	a3ss = 0.09 a5ss = 0.06	0.07		
	HCa	0.51	0.14	a3ss = 0.08 a5ss = 0.05	0.22		
SUPPA	PCa	0.37	0.08	a3ss = 0.14 a5ss = 0.14	0.006	0.23	0.04
	HCa	0.34	0.08	a3ss = 0.16 a5ss = 0.14	0.004	0.24	0.03
SUPPA2	PCa	0.38	0.08	a3ss = 0.14 a5ss = 0.13	0.004	0.22	0.04
	HCa	0.33	0.08	a3ss = 0.16 a5ss = 0.15	0.006	0.24	0.04

^1^ *ES* Exon skipping, *RI* Retained intron, *ASS* Alternative splice site, *a3ss* Alternative 3′ splice site, *a533* Alternative 5′ splice site, *MXE* Mutually exclusive event, *AF* Alternative first exon, *AL* Alternative last exon.

### Overlap of DS genes between tools

**Figure 3:**
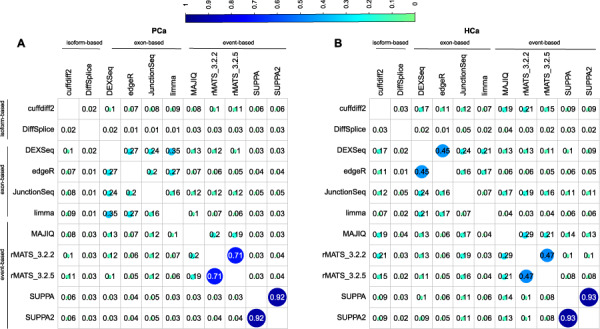
Similarity between the methods in the complete PCa and HCa data sets. Overlap of top 500 ranked DS genes between the methods in the (**A**) PCa and (**B**) HCa data set. Genes were ranked based on the FDR in all methods except for DiffSplice, which provided its own test statistic to rank the genes instead. dSpliceType was excluded from this comparison as it allowed listing genes only until FDR of 0.05 which provided less than 500 genes in both data sets.

As the different tools reported a hugely variable number of DS genes (from 0 to more than 14 000, with the median number of 1376 in the PCa and 911 in the HCa data set), we decided to focus on the top 500 ranking genes for evaluating the overlap across the tools. dSpliceType was excluded from the analysis as it did not allow reporting DS genes with FDR above 0.05 and thus provided less than 500 genes altogether. Notably, the overlap of the 500 top-ranking genes between the tools in general was strikingly low (Figure **3**). The highest overlap across the data sets was observed between the two versions of SUPPA (>92%) and between the two versions of rMATS (>47%). This shows that although the two versions of SUPPA call a different number of DS genes, the ranking of the top genes is very similar, while with rMATS there is more difference also in the ranking of the genes. Between different tools, the highest overlap was observed between the exon-based methods DEXSeq and limma (35%) in the PCa data set and DEXSeq and edgeR (45%) in the HCa data set. DEXSeq in general had highest overlap with the other exon-based tools in both data sets (>24% in PCa data set and >21% in HCa data set). Isoform-based DiffSplice had a low overlap (<10%) with all other tools in both data sets. In both data sets, cuffdiff2 had the highest overlap with rMATS_3.2.2 (10–21%) and DEXSeq (10–17%). MAJIQ overlapped highest with rMATS in both data sets (19–29%). Overlaps based on DS genes reported by each tool (after applying FDR cutoff of 0.05) are available in Supplementary Figure S4. Also dSpliceType was included in this comparison, showing its low general overlap with all other tools.

**Table 4:** Proportion of qPCR-validated DS genes and the total number of DS genes detected in the MVS and HVS data sets with the different tools

Tool	Data set	Proportion of qPCR-validated genes detected	Number of DS genes detected
cuffdiff2	MVS	0.00	0
	HVS	0.56	478
DiffSplice	MVS	0.00	0
	HVS	0.00	533
DEXSeq	MVS	0.04	209
	HVS	0.94	5833
edgeR	MVS	0	75
	HVS	0.94	6798
JunctionSeq	MVS	0.18	601
	HVS	0.69	3160
limma	MVS	0.07	831
	HVS	0.97	959
MAJIQ	MVS	0.88	1326
	HVS	0.94	1811
dSpliceType	MVS	–	–
	HVS	0.13	940
rMATS v3.2.2	MVS	0	0
	HVS	1.00	2962
rMATS v3.2.5	MVS	0.04	25
	HVS	1.00	4486
SUPPA	MVS	0.71	1433
	HVS	0.97	2706
SUPPA2	MVS	0.58	736
	HVS	0.91	1495

For the event-based tools, we also calculated the proportions of the different types of events reported among the top 500 detections from each tool, summarized in Table 3. This revealed marked differences between the tools: SUPPA/SUPPA2 mostly reported exon-skipping events. MAJIQ and rMATS reported more exon-skipping events in the HCa data set and more intron retention events in PCa data set.

### Comparison to qPCR validations

The major reason for the inclusion of the MVS and HVS data sets in this study was to bring the chosen DS analysis tools to bear on data sets wherein we have, even if only a limited number of qPCR-validated splicing events (28 in MVS and 32 in HVS data set). It should, however, be noted here that rMATS was used in the original studies to determine the splicing events selected for the qPCR validation. While all tools recovered a clearly higher proportion of validated genes in the larger HVS data set, the number of the detected, validated and total DS genes varied considerably between the different tools (Table 4). MAJIQ and SUPPA detected overall the highest proportion of the qPCR-validated DS genes across the data sets (MAJIQ 88% and SUPPA 71% in the MVS data set; MAJIQ 94% and SUPPA 97% in the HVS data set). Among the exon-based tools, limma detected the highest proportion (97%) of qPCR-validated genes in the HVS data set. Although the event-based rMATS v3.2.5 detected only 4% of the validated DS genes in the MVS data set, it was able to recover all 32 validated DS genes in the HVS data set. Isoform-based tools DiffSplice and cuffdiff2 did not detect any DS genes in the MVS data set. In the HVS data set, DiffSplice recovered none of the validated DS genes, whereas cuffdiff2 recovered 56% of the validated DS genes, which was a relatively high proportion considering it only detected 478 DS genes in total. dSpliceType did not provide any result for the MVS data set due to an unknown technical error.

### Functional enrichment analysis

**Figure 4:**
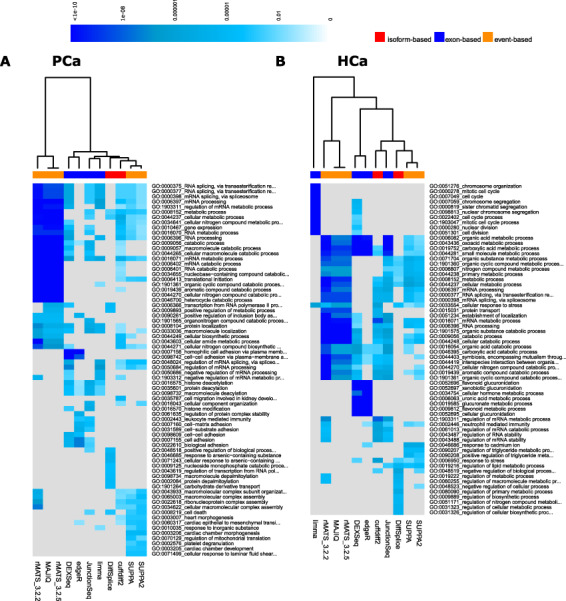
Heatmap of the *P*-values of the top enriched GO biological processes across the methods in the complete (**A**) PCa and (**B**) HCa data sets. Grey colour represents missing values.

To further investigate the DS gene lists provided by the different tools, we ran GO [48] enrichment analysis based on the top 500 ranking genes in each method, in both PCa and HCa data sets. We then collected the combined list of top 10 most enriched GO terms related to GO biological processes in each method and clustered them together based on the enrichment test *P*-value (Figure 4). The overall most significant (*P*-value <0.05) enriched biological processes found in at least nine of the tools were GO:0000375 (RNA splicing, via transesterification reaction), GO:0000377 (RNA splicing, via transesterification reaction with bulged adenosine as nucleophile), GO:0000398 (mRNA_splicing, via splicesome), GO:0006397 (mRNA processing) and GO:0016071 (mRNA metabolic process) in the PCa data set and GO:0019752 (carboxylic acid metabolic process) and GO:0044281 (small molecule metabolic process) in the HCa data set. Overall, the most significantly enriched of GO terms were detected by rMATS and MAJIQ in both data sets. These methods also clustered together according to their GO enrichment. Although this work does not focus on biological mechanisms, this preliminary result nevertheless warrants further investigation as to the DS of the splicing machinery genes themselves as drivers of cancer or other disease processes.

### Runtime and memory consumption

**Figure 5:**
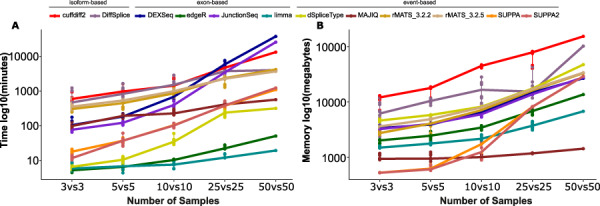
Memory usage and run time of the methods with different numbers of replicates in HCa data set. (**A**) Run time and (**B**) maximum memory required, as measured by Linux process accounting tool acct. Values are on log10 scale.

Analysis of high-throughput sequencing data is a computationally intensive task. Major measures of performance of the tools are their maximum memory consumption and total runtime with increasing numbers of samples, which were here ascertained for all tools (Figure 5). All tools were run on a computer cluster managed by the free, open-source Simple Linux Utility for Resource Management (SLURM). The runtimes reported do not include the time for producing the files that were needed for running the tools such as exon count files. Overall, limma and edgeR outperformed all other methods in terms of time, whereas MAJIQ took the least maximum memory, followed by limma and edgeR. limma and edgeR took less than an hour to run, whereas cuffdiff2, DEXSeq, JunctionSeq, rMATS and DiffSplice took days to run (Figure 5A). Of the event-based methods, dSpliceType was faster than SUPPA/SUPPA2, MAJIQ and rMATS. Of the isoform-based methods, DiffSplice was faster than cuffdiff2, even though cuffdiff2 was able to take advantage of using multiple compute cores. The tool to peak at the highest memory footprint was cuffdiff2, followed by DiffSplice (Figure 5B). Although SUPPA needed very little memory with small sample size (*n* = 3 and *n* = 5), its memory usage increased rapidly with the increasing sample size (*n* = 10, 25 and 50).

### Effect of sequencing depth

To study the effect of the sequencing depth on the performance of the methods, we compared the number of DS genes and the number of qPCR-validated genes in the HVS data set that had more than 100 million reads per sample (Supplementary Figure S5). This allowed us also to calculate the precision and recall at varying sequencing depths (downsampling from 20 to 100 million reads with increments of 20 million reads using seqtk [49] tool) using the DS genes detected in the full HVS data set as the truth set. According to the results, the number of detections and recall steadily increased with the increasing sequencing depth across the methods, while precision and detection of the qPCR validates were in most cases rather stable after 40 to 50 million reads per sample. However, for dSpliceType the number of recovered qPCR-validated genes started decreasing after 80 million reads per sample.

### Effect of differentially expressed genes

To investigate whether there were differences in the proportions of differentially expressed genes between the DS gene lists reported by the different tools, we performed gene-level differential expression analysis in the complete PCa and HCa data sets with limma requiring FDR < 0.05 and absolute fold-change >2 (Supplementary Table S6). The proportion of differentially expressed genes among the DS genes ranged from 9 to 20% in the PCa and from 23 to 28% in the HCa data set. The result suggests that the detection of DS genes was not largely affected by the differential expression status of the gene.

## Discussion

In this study, we evaluated 10 tools (Table 1) for the analysis of DS genes, representing both isoform- and count-based (exon-based and event-based) approaches, using four RNA-seq data sets (Table 2) that were selected based on the relatively large number of replicates available, sufficient read length and sequencing depth and/or availability of qPCR-validated splicing events. In the lack of comprehensive ground truth, we included in our comparison several approaches, with the assumption that methods that constantly show robust performance across the various evaluation metrics can be expected to perform overall best also in other studies. To account for potential systematic bias in the detections reported by the methods, we performed true and mock comparisons in the subsets and complete data sets. The samplings and comparisons were always repeated 10 times to avoid the chance of extreme results due to a particular sample combination. The mock comparisons were performed using samples from the same (normal) group to estimate the number of FP. Normal sample group was used, as differences between the random subsets were expected to be minor compared to differences in the tumor group, where subsets of samples are not expected to be homogeneous. Similar subsampling-based approaches have been used previously, for example in [50] and [51].

The isoform, exon and event-level results were aggregated to the gene level in order to compare the different methods (see Supplementary File for the details of the aggregation procedure for each method). Our original plan was to investigate the results also at a more detailed level of individual events and exons but the low overlap observed already at the gene level suggested that this comparison would not provide meaningful results.

In the HCa and PCa data sets, all the exon-based methods (DEXSeq, edgeR, JunctionSeq, limma) and two event-based methods (MAJIQ and rMATS) overall performed robustly, showing low FDR, high precision and moderate recall (Figure **2**). Although the overlap of top-ranking DS genes across the tools was relatively low, in general, the exon-based methods had highest overlaps with each other, while they showed least overlap with the isoform-based methods (Figure **3**). Strongest overall enrichment of GO terms was observed by the event-based methods rMATS and MAJIQ (Figure 4). MAJIQ and SUPPA scored overall best in terms of the proportion of qPCR-validated DS genes in both MVS and HVS data set, followed by SUPPA2 (Table 4). In terms of time and memory consumption, limma and edgeR clearly outperformed all other tools, while MAJIQ took the least maximum memory (Figure 5). The fact that no single tool outperformed the others across all measures is in agreement with the findings of the previous work carried out on simulated data and real plant RNA-seq data [17]. Out of the 10 tools included in our comparison, DEXSeq, rMATS, cuffdiff2 and DiffSplice were included also in this previous comparison, where DEXSeq and rMATS were in general found to perform well.

We observed that with most methods, the number of detected DS genes increased and the relative variation across the randomly sampled subsets decreased with larger numbers of replicates, as expected. However, cuffdiff2 reported very few DS genes in all but HVS data set and with dSpliceType and SUPPA, the number of reported DS genes consistently decreased when the number of replicates increased in PCa and HCa data sets. Similar poor performance (no results or decreasing number of detections with an increasing number of replicates) has been earlier shown for cuffdiff2 in previous studies both in the context of differential gene expression [50, 52, 53] and DS [51]. The studies suggest that the problem may be derived from the inability of cuffdiff2 to deal with the inherent biological variability of larger numbers of samples.

In our comparison, the tools were run using default settings, as this is how most users would in practice do. Initially, we performed all the analyses based on alignments produced using Tophat2 [54] but later revised them based on alignments produced by STAR [43] that was shown in a recent study [55] to be a robust aligner (also in regard to splice junction counts) despite the choice of the run parameters, while Tophat2 was found particularly sensitive to its parameter settings. Another recent study [56] further investigated the different parameter settings for STAR and concluded it to be very robust when run on default settings. However, the general trends reported here were observed also in the earlier Tophat2 based results (data not shown). In our study, only two groups were compared at a time. For more complex experimental setups, more sophisticated comparison designs and also incorporation of confounding variables, such as batch effects, may be needed. All exon-based methods have a modelling-based flexible support for complex experimental designs including confounding variables (Table 2). Cuffdiff2, dSpliceType and MAJIQ only support unpaired two-group comparisons while DiffSplice additionally allows one variable for blocking. rMATS and SUPPA/SUPPA2 support paired sample setups.

For all methods, we used the same complete annotation file (including definitions of gene structures), except for DiffSplice that does not use any annotation. Some of the methods (cuffdiff2, JunctionSeq, rMATS, MAJIQ) detect also unannotated novel splicing events, but we limited our comparisons to the known annotations. Considering the low overlap observed between the different methods and the fact that significant performance decrease with incomplete annotation has been shown earlier [17], comparisons based on incomplete annotations did not seem meaningful for the present study.

The effect of sequencing depth and read length in regard to DS has been studied earlier. The study by Liu et al. [17] suggested that most methods were fairly robust to different read depths or coverage of RNA-seq (25× to 100×), with a minor drop of discrimination power when the read depth decreased. Chhangawala et al. [57], on the other hand, showed that there was a marked improvement in the detection of known and novel splice sites when longer read lengths (≥100 bp) and paired-end data were used. Our analysis reconfirmed the DS results to be quite robust after 40 to 60 million reads per sample (Supplementary Figure S5). Additionally, we analyzed whether the differential expression status of the gene greatly affected the results but did not find evidence of this in our comparisons (Supplementary Table S6).

In this study, our aim was to compare methods that claim to perform DS analysis from RNA-seq data using different approaches. The different tools provide the results at the exon, event or isoform level depending on the strategy employed by them. Our comparison showed how the results vary largely across the different methods even for the same approach category. The low general overlap of the results especially across the event-based tools may, in fact, be partly explained by the large differences in the approaches taken by the different methods, which may yield to varying strength in identifying different types of splicing events, as depicted in our analysis (Table 3). While rMATS and dSpliceType for example consider an intron retention event as long as it is detected in one of the transcripts of a gene, SUPPA calculates the ratio of the abundance of transcripts that include one form of the event over the abundance of the transcripts that contain other forms of the event. The calculation of PSI values also varies across the event-based methods, making comparison of the values produced by different methods difficult. Isoform-based methods, on the other hand, may have a decreased power to detect DS, which has been earlier speculated in [58]. Supporting this observation, although cuffdiff2 and DiffSplice reported very few DS genes in our comparisons, the top-ranking genes had some overlap with those of the other tools.

Thus, our main conclusion is that running several tools is advisable in order to generate a comprehensive view of the DS among the studied samples. While the isoform-based methods compare the relative isoform abundances and exon-based methods compare exon and exon-junction read counts, the event-based methods compare the quantitated splicing events. All of the three approaches have the general goal to reveal differences in the gene expression as the outcome of the operation of the splicing machinery. Currently, it remains an open question which of these approaches (or which combination of these approaches) will prove to be most useful in elucidating the underlying biological phenomena and more application studies will be needed to answer this question.

To our knowledge, this work provides the first independent cross-comparison of DS analysis tools across real vertebrate data sets. However, it would be interesting to repeat the evaluation when more real data sets with fairly large numbers of replicates, sufficient sequencing depth and read length and/or more qPCR-validated DS genes becomes available.

## Conclusions

In our comparison, all the exon-based methods (DEXSeq, edgeR, JunctionSeq, limma) and the two event-based methods, MAJIQ and rMATS, overall performed well in terms of the number of detections, as well as precision and recall in detecting DS across the four data sets analysed. These methods also achieved a moderate FDR and recovered a reasonably high proportion of the previously qPCR-validated DS genes, thus presenting these tools as the currently best available candidates for DS analysis in RNA-seq data. In practice, the limitation of rMATS in requiring equal read length across the input data may need to be considered. Where computational performance is a concern (maximum memory and run time), limma and edgeR are recommended over the other tools. However, currently we would recommend running DS analysis using more than one tool due to the relatively large variability of the results reported by the different tools.

## Authors’ contribution

A.M. performed the analysis and wrote the manuscript. A.L. and M.V. participated in writing the manuscript and in supervising the study. A.J.M. participated in writing the manuscript. N.W. participated in performing the analysis. L.L.E. conceived and supervised the study and participated in writing the manuscript.

## Supplementary Material

Supplementary_Figures_bbz126

Supplementary_Tables_bbz126

Supplementary_File_bbz126

## Notes

Key Points

- Several computational tools have been developed to identify differentially spliced genes from RNA-seq data but a systematic evaluation of their performance on higher eukaryotes has been lacking.
- Highly variable performance of the different methodological approaches and tools were observed in the detection of DS in terms of consistency, reproducibility, running time and memory usage.
- The exon-based tools generally performed better than isoform and event-based tools.
- All the exon-based tools (limma, edgeR, DEXSeq and JunctionSeq) and two event-based methods, MAJIQ and rMATS, showed overall best performance. limma and edgeR are recommended when computational performance is a concern.

## Notes

**Arfa Mehmood** is a doctoral student in bioinformatics at the University of Turku, Finland. Her research focuses on differential splicing analysis using RNA-sequencing data under the supervision of Professor Laura L. Elo.

**Asta Laiho** is a bioinformatician at the Medical Bioinformatics Centre at the University of Turku, Finland. She has a strong background in the analysis of high-throughput sequencing data.

**Mikko S. Venäläinen** is a postdoctoral researcher at the Medical Bioinformatics Centre at the University of Turku, Finland. He is an expert in solving biomedical problems using machine learning approaches.

**Aidan J. McGlinchey** was a postdoctoral researcher in bioinformatics at the Medical Bioinformatics Centre at the University of Turku, Finland. Currently, he works as a postdoctoral research associate at Örebro University, Sweden.

**Ning Wang** is a doctoral student in bioinformatics at the University of Turku supervised by Professor Laura Elo.

**Laura L. Elo** is a professor of computational medicine, research director in bioinformatics and head of the Medical Bioinformatics Centre at the Turku Bioscience Centre, University of Turku and Åbo Akademi University, Turku, Finland. Her main research interests include computational biomedicine and bioinformatics.
