# Supplementary material for: Systematic evaluation of differential splicing tools for RNA-seq studies
Source: Brief Bioinform. 2019 Dec 5;21(6):2052–65. doi: 10.1093/bib/bbz126 (PMC7711265; doi:10.1093/bib/bbz126)
Supplement: Supplementary_Figures_bbz126 [file supplementary_figures_bbz126.docx]

**Figure S1.** Schematic illustration of the methodologies developed for differential splicing (DS) analysis of RNA-sequencing data. The methods highlighted in grey do not support differential splicing testing of replicate samples. The methods represented in bold were selected for comparison in this study.


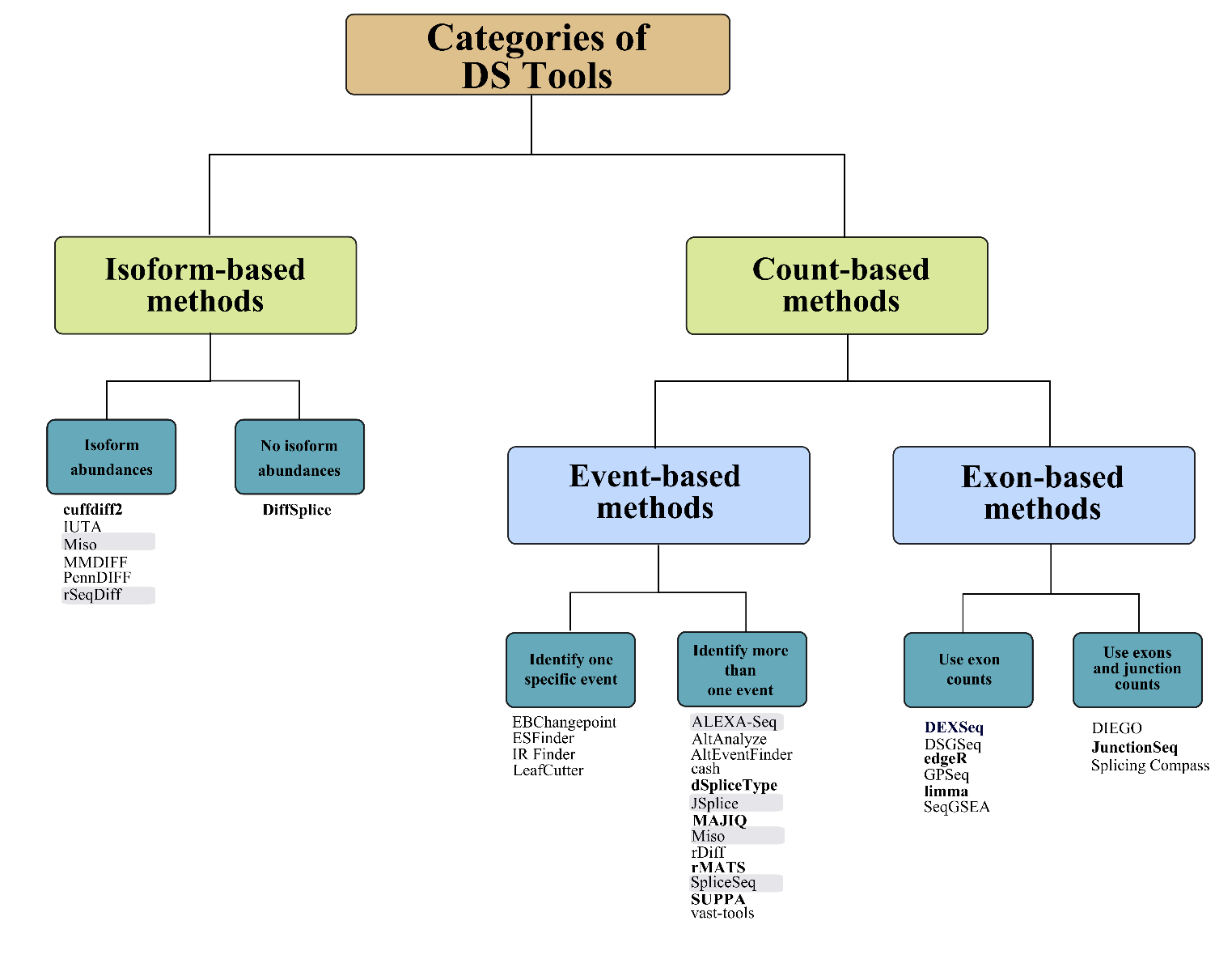


**Figure S2.** Principal Component Analysis (PCA) plots of log2 transformed count per million (CPM) values of genes in **(A)** PCa **(B)** HCa **(C)** HVS, and **(D)** MVS datasets.

**
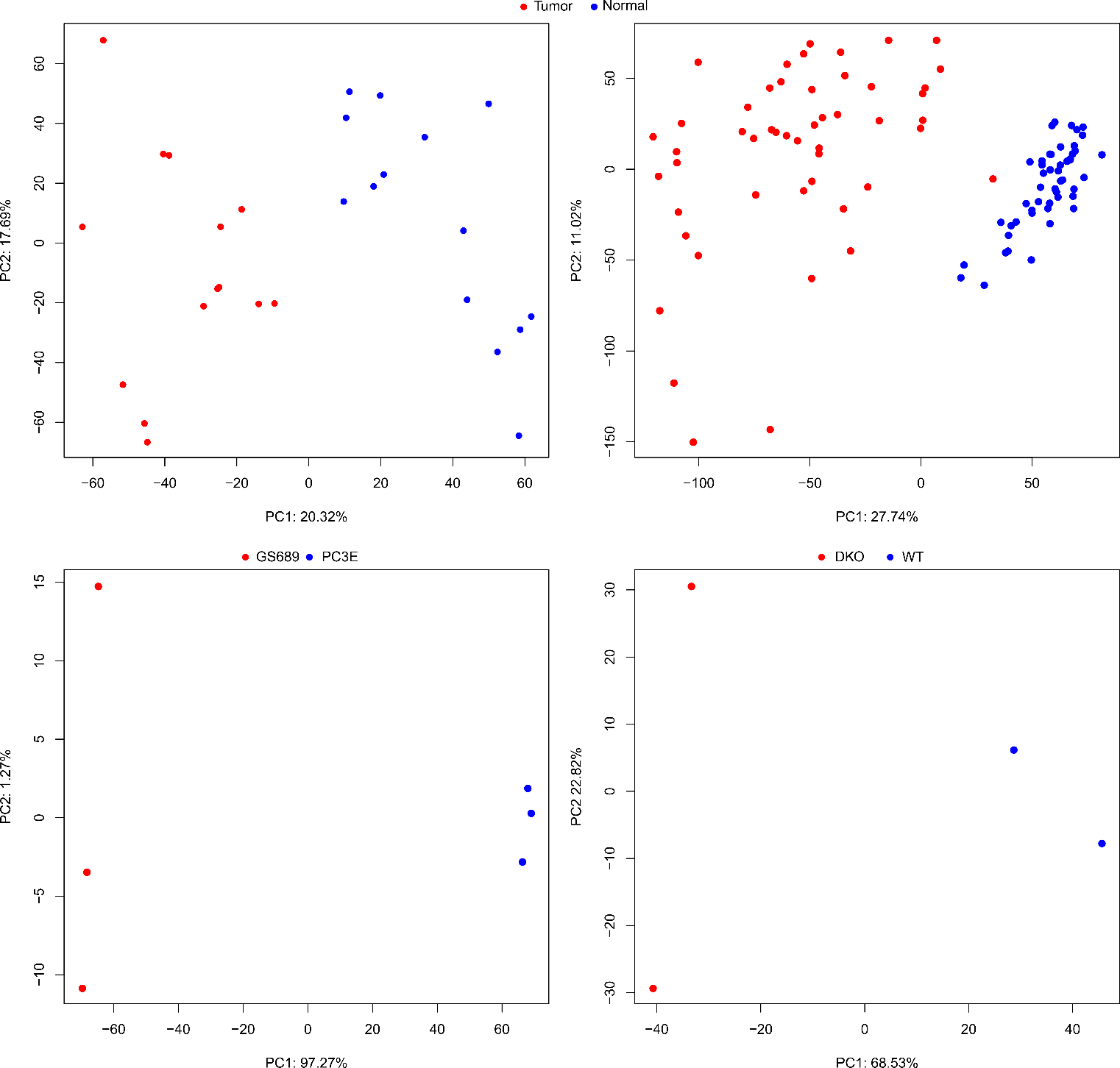
**

**Figure S3.** Number of detections, precision, recall and false discovery rate of the ten compared methods in PCa and HCa datasets with different numbers of replicates. Number of differentially spliced (DS) genes in the **(A)** PCa and **(B)** HCa dataset. Precision of the tools in the **(C)** PCa and **(D)** HCa dataset. Recall of the tools in the **(E)** PCa and **(F)** HCa dataset. False discovery rate of the tools in the **(G)** PCa and **(H)** HCa dataset. DS genes were detected at a false discovery rate of 0.05 between the tumor and normal condition. The points in the boxplots correspond to the ten random subsets; in the complete dataset the analysis was performed once. In precision and recall, the values were calculated by considering the DS genes detected in the complete dataset as true. For the false discovery rate, the number of detections in the artificial mock comparisons within the normal sample group were divided by the median number of detections in the real comparisons with the same number of samples. In some cases, there were more detections from the mock comparisons than the corresponding real comparison, in which case the false discovery rates were truncated to 1.


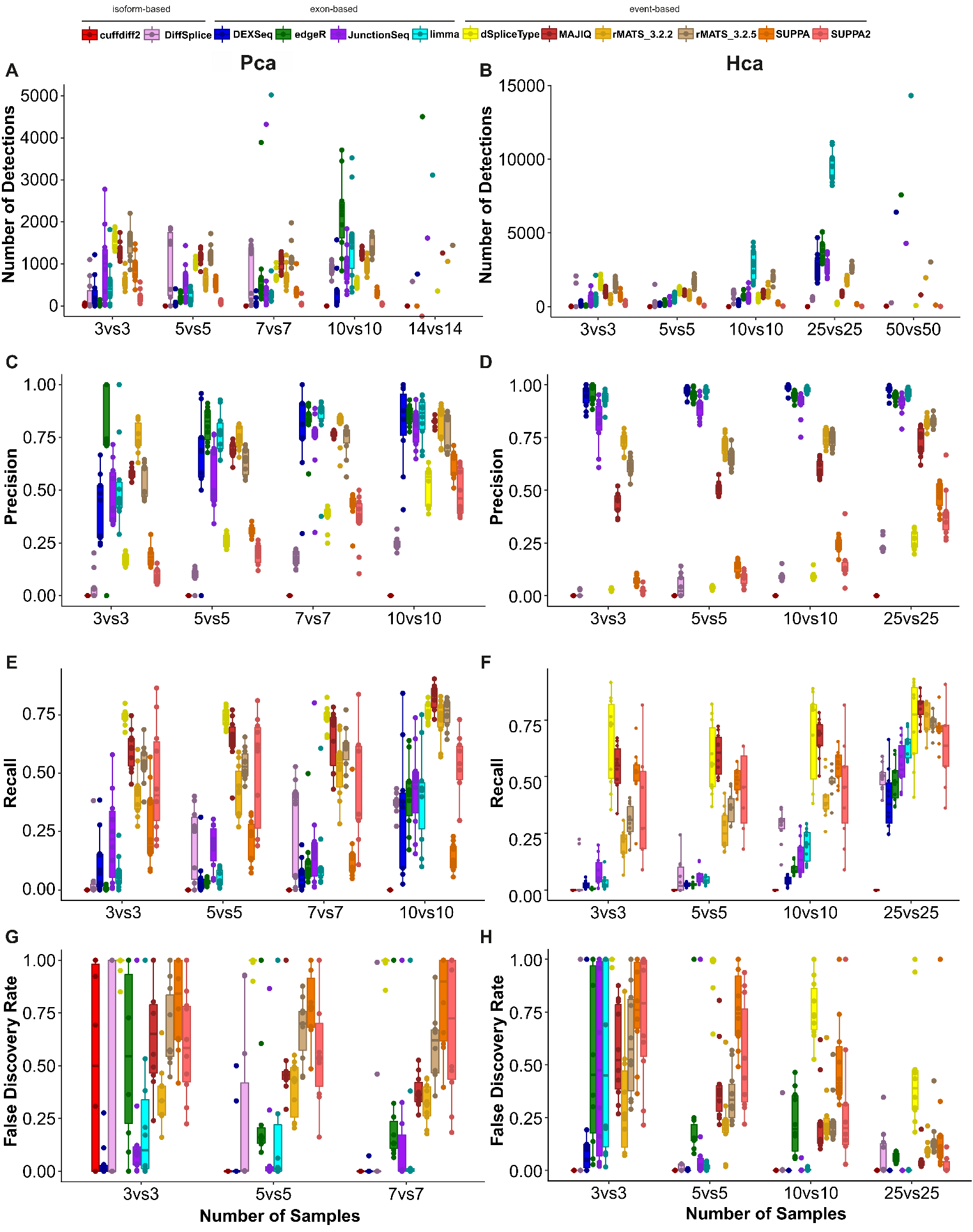


**Figure S4.** Similarity between the methods. Overlap of differentially spliced genes (FDR < 0.05) in the complete **(A)** PCa and **(B)** HCa datasets.


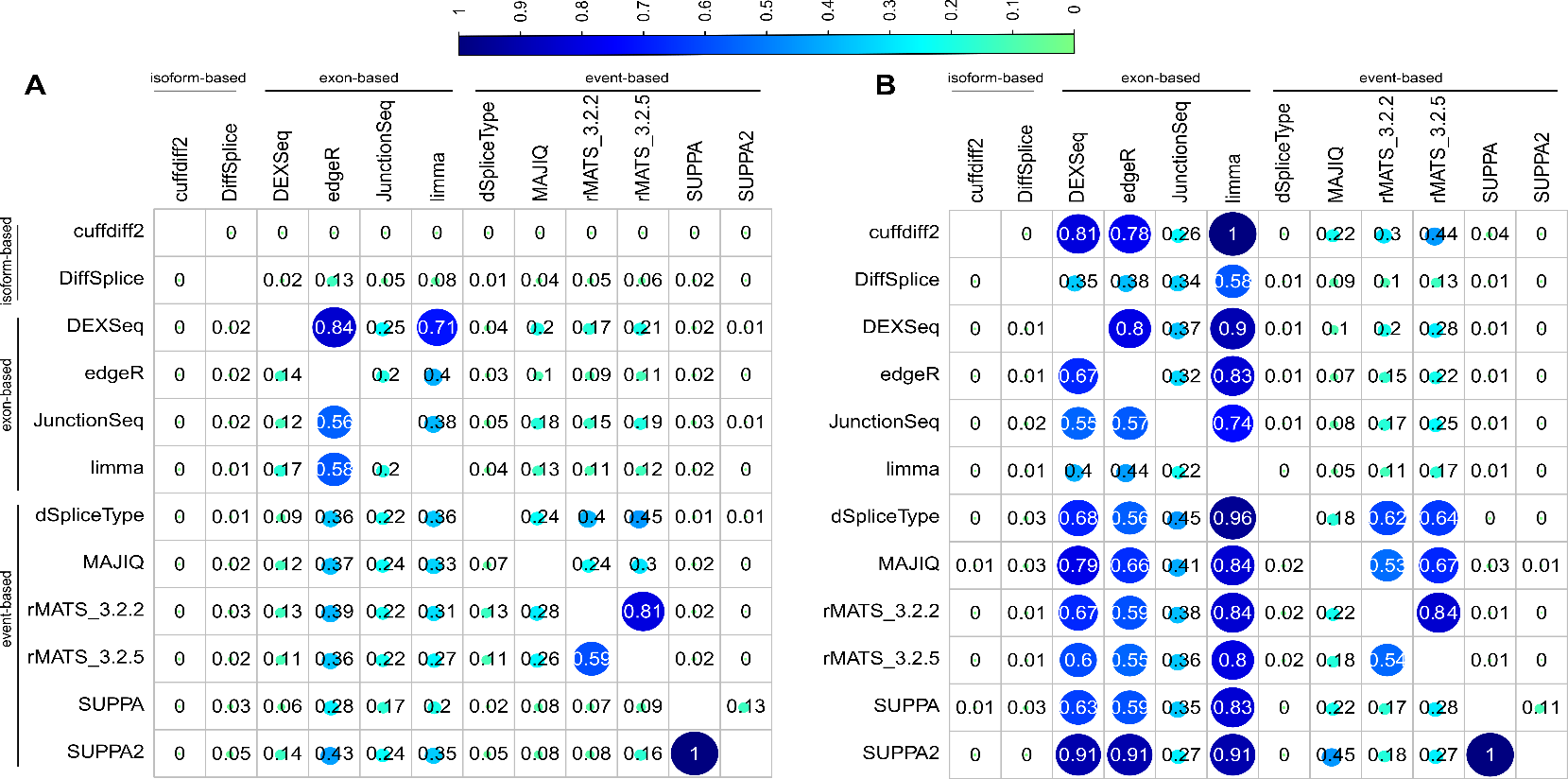


**Figure S5.** Number of detections, precision, recall and overlap with qPCR validated DS genes of the ten compared methods with sequencing depth of 20, 40, 60, 80, and 100 million reads and the original full HVS dataset. **(A)** Number of differently spliced (DS) genes found at different sequencing depths. **(B)** Precision and **(C)** Recall of the tools at different sequencing depths by considering the DS genes in the original HVS dataset as the truth set. **(D)** The number of qPCR validated DS genes found by each tool at different sequencing depths.

**
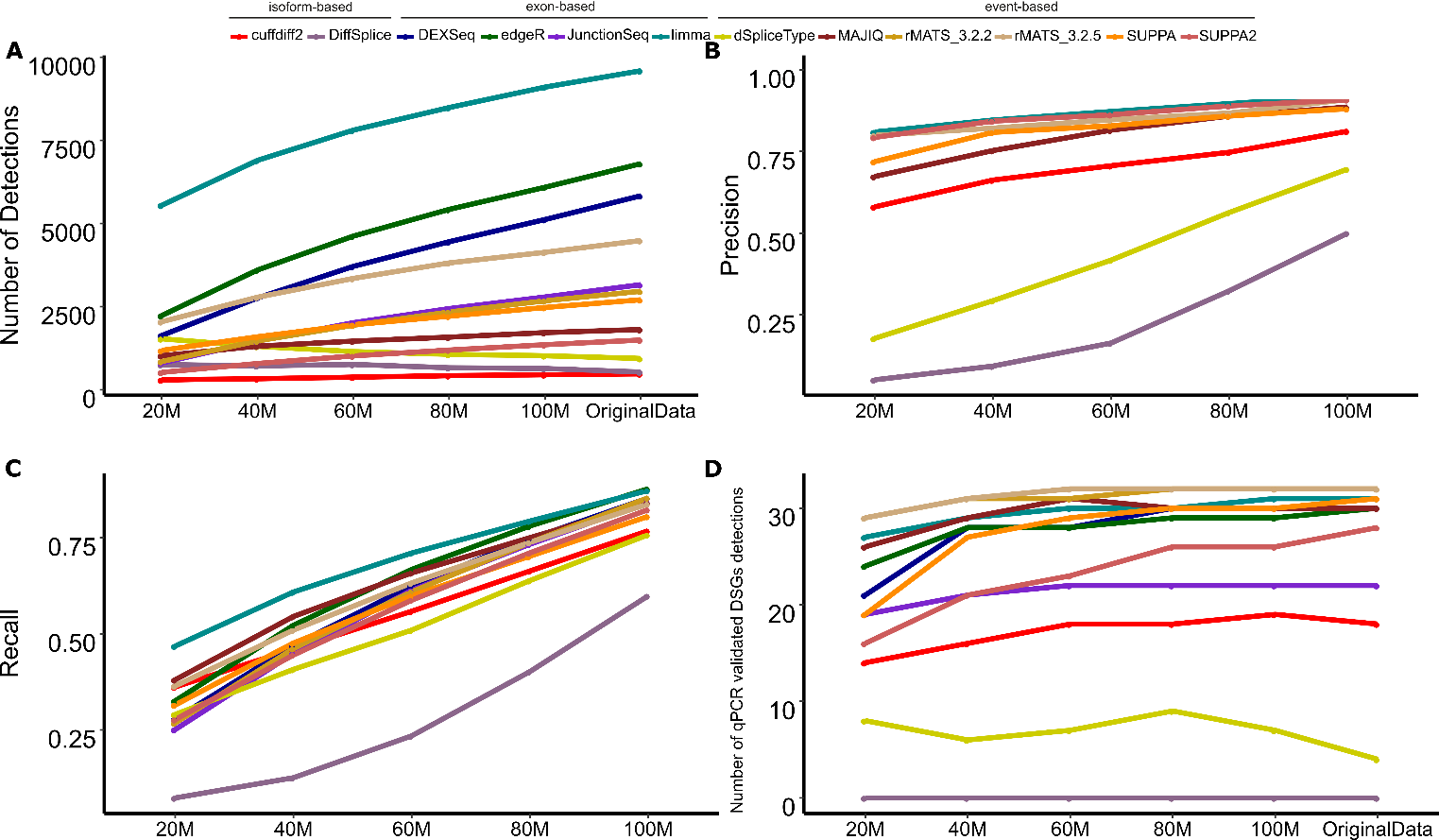
**
