# Supplementary material for: Systematic evaluation of differential splicing tools for RNA-seq studies
Source: Brief Bioinform. 2019 Dec 5;21(6):2052–65. doi: 10.1093/bib/bbz126 (PMC7711265; doi:10.1093/bib/bbz126)
Supplement: Supplementary_Tables_bbz126 [file supplementary_tables_bbz126.docx]

**Table S1.** Mapping statistics of the samples in the PCa, HCa, MVS, and HVS datasets.

| Dataset | Mean number of total reads | Range of total number of reads | Mean number of mapped reads | Range of number of mapped reads |
| --- | --- | --- | --- | --- |
| PCa | 67360864 | 60842900 **-** 75144440 | 62862896 | 55851622**-**  69272664 |
| HCa | 48801207 | 30677604 - 75675538 | 45281324 | 27358430 -  71672294 |
| MVS | 111560528 | 78601692 - 166191916 | 97134186 | 67085056 - 148058214 |
| HVS | 248731591 | 228341994-264673120 | 230163514 | 210071934 - 243946800 |

**Table S2.** Median and standard error of the number of detections in the ten random subsets with different sample sizes (rows) in the PCa and HCa datasets.

##

|  |  | n | 1 | 2 | 3 | 4 | 5 | 6 | 7 | 8 | 9 | 10 | 11 | 12 |
| --- | --- | --- | --- | --- | --- | --- | --- | --- | --- | --- | --- | --- | --- | --- |
| Number of Detections | **PCa** | **3** | 13 (7.73) | 1 (125.43) | 127 (126.94) | 11 (17.8) | 1013 (288.44) | 404 (169.52) | 1587 (69.31) | 1292 (66.89) | 586 (60.26) | 1578 (122.29) | 925 (104.92) | 200 (50.08) |
|  |  | **5** | 0 (0) | 1490 (254.88) | 12 (39.43) | 179 (28.72) | 625 (128.24) | 162 (48.71) | 999 (30.8) | 1208 (54.86) | 591 (50.89) | 1203 (70.03) | 563 (42.07) | 110 (13.06) |
|  |  | **7** | 0 (0) | 1301 (211.27) | 41 (36.31) | 448 (351.24) | 390 (407.88) | 300 (475.09) | 664 (44.78) | 1163 (65.43) | 682 (79.75) | 1124 (103.68) | 355 (70.88) | 46 (26.03) |
|  |  | **10** | 0 (0) | 890 (47.62) | 334 (153.09) | 2265 (289.83) | 932 (151.84) | 1572 (327.69) | 491 (30.39) | 1259 (31.76) | 942 (66.09) | 1324 (84.86) | 305 (24.18) | 36 (5.76) |
|  |  | **14** | 0 (0) | 583 (0) | 761 (0) | 4506 (0) | 1616 (0) | 3115 (0) | 354 (0) | 1259 (0) | 1060 (0) | 1444 (0) | 288 (0) | 47 (0) |
|  | **HCa** | **3** | 0 (2.82) | 1 (247.14) | 162 (38.52) | 37 (10.36) | 260 (143.01) | 313 (192.71) | 1552 (108.97) | 976 (74.38) | 584 (53.95) | 1450 (100.07) | 724 (77.25) | 135 (31.81) |
|  |  | **5** | 0 (0) | 33 (146.88) | 121 (20.28) | 177 (34.49) | 252 (65.89) | 590 (80.7) | 1125 (62.19) | 887 (45.78) | 711 (71.26) | 1659 (101.58) | 367 (26.47) | 64 (7.21) |
|  |  | **10** | 0 (0) | 919 (86.33) | 241 (43.01) | 644 (73.83) | 603 (142.74) | 2931 (340.23) | 601 (54.58) | 907 (46.96) | 993 (91.49) | 1898 (89.26) | 238 (17.2) | 35 (4.42) |
|  |  | **25** | 0 (0) | 558 (22.48) | 2493 (309.78) | 3800 (251.85) | 2448 (199.71) | 9514 (324.31) | 182 (20.7) | 869 (42.02) | 1879 (69.17) | 2714 (66.2) | 150 (9.04) | 18 (2.81) |
|  |  | **50** | 27 (0) | 257 (0) | 6406 (0) | 7571 (0) | 4289 (0) | 14313 (0) | 73 (0) | 794 (0) | 1958 (0) | 3025 (0) | 102 (0) | 11 (0) |

## Columns: n = Number of samples in each sample group, 1 = cuffdiff2, 2 = DiffSplice, 3 = DEXSeq, 4 = edgeR, 5 = JunctionSeq, 6 = limma, 7 = dSpliceType, 8 = MAJIQ, 9 = rMATS_3.2.2, 10 = rMATS_3.2.5, 11 = SUPPA, 12 = SUPPA2

##

**Table S3.** Median and standard error of the precision in the ten random subsets with different sample sizes (rows) in the PCa and HCa datasets.

|  |  | n | 1 | 2 | 3 | 4 | 5 | 6 | 7 | 8 | 9 | 10 | 11 | 12 |
| --- | --- | --- | --- | --- | --- | --- | --- | --- | --- | --- | --- | --- | --- | --- |
| Precision | **PCa** | **3** | 0 (0) | 0 (0.02) | 0.37 (0.06) | 0.73 (0.12) | 0.41 (0.05) | 0.46 (0.07) | 0.17 (0.01) | 0.57 (0.01) | 0.73 (0.02) | 0.49 (0.02) | 0.18 (0.02) | 0.08 (0.01) |
|  |  | **5** | 0 (0) | 0.1 (0.02) | 0.69 (0.09) | 0.83 (0.03) | 0.49 (0.05) | 0.77 (0.03) | 0.26 (0.01) | 0.69 (0.01) | 0.75 (0.02) | 0.65 (0.02) | 0.31 (0.01) | 0.21 (0.02) |
|  |  | **7** | 0 (0) | 0.17 (0.01) | 0.78 (0.1) | 0.83 (0.03) | 0.77 (0.05) | 0.88 (0.05) | 0.4 (0.02) | 0.76 (0) | 0.82 (0.02) | 0.77 (0.02) | 0.44 (0.02) | 0.37 (0.04) |
|  |  | **10** | 0 (0) | 0.24 (0.01) | 0.84 (0.06) | 0.86 (0.02) | 0.82 (0.03) | 0.87 (0.03) | 0.56 (0.03) | 0.82 (0.01) | 0.87 (0.03) | 0.83 (0.03) | 0.66 (0.02) | 0.46 (0.04) |
|  | **HCa** | **3** | 0 (0) | 0 (0) | 0.95 (0.02) | 0.99 (0.01) | 0.83 (0.04) | 0.94 (0.01) | 0.03 (0) | 0.45 (0.02) | 0.73 (0.01) | 0.62 (0.01) | 0.07 (0.01) | 0.02 (0.01) |
|  |  | **5** | 0 (0) | 0.02 (0.02) | 0.97 (0.01) | 0.96 (0.01) | 0.91 (0.02) | 0.97 (0.01) | 0.04 (0) | 0.51 (0.01) | 0.71 (0.02) | 0.65 (0.02) | 0.14 (0.01) | 0.08 (0.01) |
|  |  | **10** | 0 (0) | 0.09 (0.01) | 0.99 (0.01) | 0.95 (0.01) | 0.92 (0.02) | 0.98 (0) | 0.09 (0.01) | 0.59 (0.01) | 0.76 (0.01) | 0.76 (0.01) | 0.24 (0.01) | 0.12 (0.03) |
|  |  | **25** | 0 (0) | 0.22 (0.01) | 0.98 (0.01) | 0.94 (0.01) | 0.93 (0.02) | 0.96 (0.01) | 0.27 (0.01) | 0.76 (0.02) | 0.81 (0.01) | 0.83 (0.01) | 0.49 (0.02) | 0.38 (0.04) |

## Columns: n = Number of samples in each sample group, 1 = cuffdiff2, 2 = DiffSplice, 3 = DEXSeq, 4 = edgeR, 5 = JunctionSeq, 6 = limma 7 = dSpliceType, 8 = MAJIQ, 9 = rMATS_3.2.2, 10, rMATS_3.2.5, 11 = SUPPA, 12 = SUPPA2

**Table S4.** Median and standard error of the recall in the ten random subsets with different sample sizes (rows) in the PCa and HCa datasets.

|  |  | n | 1 | 2 | 3 | 4 | 5 | 6 | 7 | 8 | 9 | 10 | 11 | 12 |
| --- | --- | --- | --- | --- | --- | --- | --- | --- | --- | --- | --- | --- | --- | --- |
| Recall | **PCa** | **3** | 0 (0) | 0 (0.04) | 0.08 (0.04) | 0 (0) | 0.22 (0.06) | 0.06 (0.02) | 0.74 (0.01) | 0.61 (0.03) | 0.42 (0.03) | 0.54 (0.03) | 0.2 (0.05) | 0.41 (0.07) |
|  |  | **5** | 0 (0) | 0.26 (0.05) | 0.01 (0.03) | 0.03 (0) | 0.2 (0.03) | 0.04 (0.01) | 0.74 (0.01) | 0.65 (0.03) | 0.42 (0.04) | 0.53 (0.02) | 0.23 (0.03) | 0.61 (0.08) |
|  |  | **7** | 0 (0) | 0.38 (0.07) | 0.04 (0.02) | 0.09 (0.04) | 0.17 (0.07) | 0.09 (0.05) | 0.74 (0.02) | 0.71 (0.04) | 0.54 (0.04) | 0.61 (0.03) | 0.14 (0.04) | 0.59 (0.07) |
|  |  | **10** | 0 (0) | 0.37 (0.01) | 0.36 (0.09) | 0.42 (0.05) | 0.47 (0.06) | 0.43 (0.07) | 0.76 (0.01) | 0.84 (0.02) | 0.74 (0.03) | 0.77 (0.02) | 0.12 (0.02) | 0.54 (0.04) |
|  | **HCa** | **3** | 0 (0) | 0 (0.03) | 0.02 (0.01) | 0 (0) | 0.05 (0.02) | 0.02 (0.01) | 0.73 (0.06) | 0.55 (0.03) | 0.23 (0.02) | 0.31 (0.02) | 0.52 (0.04) | 0.27 (0.07) |
|  |  | **5** | 0 (0) | 0.02 (0.03) | 0.02 (0) | 0.02 (0) | 0.05 (0.01) | 0.04 (0.01) | 0.55 (0.05) | 0.61 (0.03) | 0.25 (0.03) | 0.36 (0.02) | 0.49 (0.02) | 0.45 (0.05) |
|  |  | **10** | 0 (0) | 0.31 (0.02) | 0.04 (0.01) | 0.08 (0.01) | 0.13 (0.03) | 0.2 (0.02) | 0.79 (0.06) | 0.69 (0.03) | 0.38 (0.03) | 0.48 (0.02) | 0.55 (0.02) | 0.45 (0.07) |
|  |  | **25** | 0 (0) | 0.5 (0.02) | 0.38 (0.04) | 0.48 (0.03) | 0.53 (0.03) | 0.65 (0.02) | 0.77 (0.06) | 0.82 (0.02) | 0.77 (0.02) | 0.74 (0.01) | 0.72 (0.02) | 0.64 (0.05) |

##

## Columns: n = Number of samples in each sample group, 1 = cuffdiff2, 2 = DiffSplice, 3 = DEXSeq, 4 = edgeR, 5 = JunctionSeq, 6 = limma, 7 = dSpliceType, 8 = MAJIQ, 9 = rMATS_3.2.2, 10 = rMATS_3.2.5, 11 = SUPPA, 12 = SUPPA2

**Table S5.** Median and standard error of the false discovery rate in the ten random subsets with different sample sizes in the PCa and HCa datasets.

|  |  | n | 1 | 2 | 3 | 4 | 5 | 6 | 7 | 8 | 9 | 10 | 11 | 12 |
| --- | --- | --- | --- | --- | --- | --- | --- | --- | --- | --- | --- | --- | --- | --- |
| False Discovery Rate | **PCa** | **3** | 0 (0) | 0 (0.04) | 0.08 (0.04) | 0 (0) | 0.22 (0.06) | 0.06 (0.02) | 0.74 (0.01) | 0.61 (0.03) | 0.42 (0.03) | 0.54 (0.03) | 0.2 (0.05) | 0.41 (0.07) |
|  |  | **5** | 0 (0) | 0.26 (0.05) | 0.01 (0.03) | 0.03 (0) | 0.2 (0.03) | 0.04 (0.01) | 0.74 (0.01) | 0.65 (0.03) | 0.42 (0.04) | 0.53 (0.02) | 0.23 (0.03) | 0.61 (0.08) |
|  |  | **7** | 0 (0) | 0.38 (0.07) | 0.04 (0.02) | 0.09 (0.04) | 0.17 (0.07) | 0.09 (0.05) | 0.74 (0.02) | 0.71 (0.04) | 0.54 (0.04) | 0.61 (0.03) | 0.14 (0.04) | 0.59 (0.07) |
|  |  | **10** | 0 (0) | 0 (0.1) | 0.11 (0.09) | 0.45 (0.13) | 0.48 (0.14) | 0.45 (0.14) | 1 (0) | 0.52 (0.07) | 0.26 (0.08) | 0.57 (0.08) | 0.76 (0.07) | 0.79 (0.1) |
|  | **HCa** | **3** | 0 (0) | 0 (0) | 0 (0) | 0.15 (0.09) | 0.01 (0.1) | 0.01 (0) | 1 (0.04) | 0.37 (0.06) | 0.23 (0.05) | 0.33 (0.04) | 0.74 (0.06) | 0.44 (0.08) |
|  |  | **5** | 0 (0) | 0 (0.04) | 0 (0) | 0.2 (0.05) | 0 (0.01) | 0 (0) | 0.73 (0.05) | 0.14 (0.06) | 0.2 (0.05) | 0.21 (0.04) | 0.41 (0.07) | 0.2 (0.09) |
|  |  | **10** | 0 (0) | 0 (0.04) | 0 (0) | 0.06 (0.01) | 0 (0) | 0 (0) | 0.39 (0.09) | 0.03 (0.02) | 0.09 (0.01) | 0.12 (0.03) | 0.1 (0.09) | 0 (0.01) |
|  |  | **25** | 0 (0) | 0 (0.04) | 0.08 (0.04) | 0 (0) | 0.22 (0.06) | 0.06 (0.02) | 0.74 (0.01) | 0.61 (0.03) | 0.42 (0.03) | 0.54 (0.03) | 0.2 (0.05) | 0.41 (0.07) |

## Columns: n = Number of samples in each sample group, 1 = cuffdiff2, 2 = DiffSplice, 3 = DEXSeq, 4 = edgeR, 5 = JunctionSeq, 6 = limma, 7 = dSpliceType, 8 = MAJIQ, 9 = rMATS_3.2.2, 10 = rMATS_3.2.5, 11 = SUPPA, 12 = SUPPA2

**Table S6.** Proportion of differentially expressed (DE) genes among the differentially spliced (DS) genes detected by the exon-based methods (DEXSeq, edgeR, JunctionSeq, limma) in the PCa and HCa datasets.

|  | PCa (DE genes = 3345 ) | | HCa (DE genes = 8025) | |
| --- | --- | --- | --- | --- |
|  | **DS genes** | **Proportion of DS genes also DE** | **DS genes** | **Proportion of DS genes also DE** |
| DEXSeq | 761 | 0.12 | 6406 | 0.23 |
| edgeR | 4506 | 0.10 | 7571 | 0.34 |
| JunctionSeq | 1616 | 0.09 | 4289 | 0.27 |
| limma | 3115 | 0.20 | 14313 | 0.28 |
