# Supplementary material for: Systematic evaluation of differential splicing tools for RNA-seq studies
Source: Brief Bioinform. 2019 Dec 5;21(6):2052–65. doi: 10.1093/bib/bbz126 (PMC7711265; doi:10.1093/bib/bbz126)
Supplement: Supplementary_File_bbz126 [file supplementary_file_bbz126.docx]

## **Execution of differential splicing (DS) tools**

**Cufflinks/cuffdiff2**

In the cufflinks/cuffdiff2 [1,2] pipeline, the isoform transcript abundances were calculated for each sample using the STAR [3] alignment bam files and Ensembl reference file (gtf format). The gtf file with the gene structure information (including information of isoforms and exons) was provided to the tool. Cufflinks was run with the following command:

cufflinks --GTF <gtf_file> --output-dir <output_dir> <alignment file>

After this, the transcript abundances across the different samples were merged using the cuffmerge function and DS analysis was finally performed using cuffdiff2. The commands used are as follows:

cuffmerge -g <gtf_file> -s <genome_fastafile> –o <output_dir> -p 8 <assembly_file.txt>

cuffdiff -o <output_dir> -p 8 -u <merged.gtf> -L group1,group2 samples_in_group1 samples_in_group2

cuffdiff2 was run using multiple computer cores. The R/Bioconductor package cummerbund [4] was used to extract the DS genes from the cuffdiff2 results.

**Limma, edgeR, DEXSeq**

To run limma [5], edgeR [6], and DEXSeq [7] splicing analysis, the Ensembl derived reference file (gff format) and the exon counts from alignment bam files were prepared using the python scripts provided with the DEXSeq package. The gff file produced takes into account that an exon can be shared by two or more genes (aggregate genes). The gff file was prepared based on the Ensembl derived gtf annotation file using the following commands:

python dexseq_prepare_annotation.py genes.gtf genes.gff

python dexseq_count.py –s <strandness> –p yes –f bam –r pos genes.gff <input.bam> <exon-counts.txt>

For limma and edgeR, the exon counts were normalized using Trimmed Mean of M-values (TMM). For limma, the normalized counts were further voom transformed [8] and then provided to the diffsplice function of the limma R/Bioconductor package. In edgeR, the normalized counts were fitted using the generalized linear model and the fit was provided to diffSpliceDGE function in the edgeR R/Bioconductor package. In DEXSeq, the exon-count data was normalized using the estimateSizeFactors function. Differential exon testing was performed using testForDEU function and the FDR values for each gene were retrieved using perGeneQValue function. For aggregate genes, the same FDR value was recorded separately for each of the genes.

**JuntionSeq**

For JunctionSeq [9], junction counts along with exon counts and the Ensembl derived reference file (gtf format) were required as input. The required files were produced using QoRTsPipeline [10] with the following command:

java –jar QoRTs.jar QC –noGzipOutput –numThreads 8 --maxReadLength <readlength> --keepMultiMapped <input.bam> <genes.gtf> <output_dir>

The resulting exon and junction count files were then provided to the R/Bioconductor package JunctionSeq function runJunctionSeqAnalyses for DS analysis. JunctionSeq produced a file for the gene-level results.

The functionality for multithreading is described in JunctionSeq’s manual but we could not get it working with our SLURM job scheduler.

**DiffSplice**

DiffSplice [11] takes alignment files in SAM format as input. The program was run using the following command:

diffsplice <settings.cfg> <datafile.cfg> <output_dir> > <diffsplice.log>

The parameter thresh_junction_filter_max_read_support was set to 2 following the procedure used in a previous comparison [12]. The gene-level results were obtained by mapping the chromosomal locations, provided by the tool, to the Ensembl derived gene annotations using biomaRt [13] R/Bioconductor package. If more than one gene matched the chromosomal location, the same result was assigned to all matching genes.

**dSpliceType**

For dSpliceType [14], the junction alignments provided by STAR were converted to bedgraph format using bedtools2 [15]. An Ensembl derived reference file was provided in gtf format and transformed to gff format using a script included in the package. The commands for running dSpliceType were as follows:

perl ensemble_gtf_to_gff.pl <genes.gtf> > <genes.gff>

bedtools genomecov –ibam <alignment.bam> -bg > <sample.bedgraph>

java -jar dSpliceType.jar -g <genes.gff> -b1 <samples_group1.bedgraph> -b2 <samples_group2.bedgraph> -j1 <junctions_group1.bed> -j2 <junctions_group2.bed> -o <output_dir>

**MAJIQ**

MAJIQ v2 takes the alignment files and the Ensembl derived reference file (gff3 format) as input. The alignment .bam files were indexed using samtools index utility. MAJIQ build was run as follows:

majiq build --disable-denovo <genes,gff3> -c <settings.ini> -j 8 –o <output_dir>

The deltapsi function was run to call DS genes:

majiq deltapsi -grp1 <samples_group1.majiq> -grp2 <sample_group2.majiq> -j 8 –o <output_dir> -n <samplegroup_name>

The voila function was then run to transform the output into human readable format:

voila tsv --threshold 0.1 <deltapsi.voila> <splicegraph.sql> -f voila.tsv

MAJIQ was run using multiple computer cores.

**rMATS**

rMATS [16] was run based on alignment .bam files and the Ensembl derived reference file (gtf format) with the following command:

python RNASeq-MATS.py -b1 <samples_group1.bam> -b2 <samples_group2.bam> -t paired -len <readlength> -gtf <genes.gtf> -c 0.0001 -analysis U -libType <librarytype> -o <output_dir>

**SUPPA**

For SUPPA [17], in order to calculate the required PSI values, transcript per million (TPM) values were calculated first using the RSEM v1.3.0 [18] tool as follows:

rsem-prepare-reference -p 8 --bowtie2 --bowtie2-path <bowtie2_path> <transcript.fa> <output_dir>

rsem-calculate-expression -p 8 --paired-end --no-bam-output --bowtie2 --bowtie2-path <bowtie2 path> --estimate-rspd <read1.fastq> <read2.fastq> <index_folder> <output_dir>

The splicing events were extracted from the Ensembl derived reference file (gtf format) and the PSI values of all the events were calculated using the following commands:

python3 suppa.py generateEvents -i <genes.gtf> -o <output> -e <list-of-events>

python3 suppa.py psiPerEvent -e <input.tpm> -i <annotation.events.ioe> -o <output_dir>

Finally, the TPM and PSI results were joined using the joinFiles option in the SUPPA pipeline and the DS testing was performed using the following command:

python3 suppa.py diffSplice --method empirical -gc --tpm <group1.tpm> <group2.tpm> --psi <group1.psi> <group2.psi> --input <annotation.events.ioe> -o <output_dir>

For running SUPPA2 the following command was used:

python3 suppa.py diffSplice --method empirical -gc –e <group1.tpm> <group2.tpm> --psi <group1.psi> <group2.psi> -i <annotation.events.ioe> -o <output_dir>

For all the event-based methods, for the gene-level comparisons, the gene was considered only once with the minimum FDR of all the splicing events related to the gene.

## **References**

1. Trapnell C, Williams BA, Pertea G, et al. Transcript assembly and quantification by RNA-Seq reveals unannotated transcripts and isoform switching during cell differentiation. Nature Biotechnology 2010; 28:511–515

2. Trapnell C, Hendrickson DG, Sauvageau M, et al. Differential analysis of gene regulation at transcript resolution with RNA-seq. Nature Biotechnology 2012; 31:46–53

3. Trapnell C, Pachter L, Salzberg SL. TopHat: Discovering splice junctions with RNA-Seq. Bioinformatics 2009; 25:1105–1111

4. Goff LA, Trapnell C, Kelley D. cummeRbund: Analysis, exploration, manipulation, and visualization of Cufflinks high-throughput sequencing data. R Package Version 22 2012;

5. Ritchie ME, Phipson B, Wu D, et al. limma powers differential expression analyses for RNA-sequencing and microarray studies. Nucleic acids research 2015; 43:e47

6. Robinson MD, McCarthy DJ, Smyth GK. edgeR: a Bioconductor package for differential expression analysis of digital gene expression data. Bioinformatics (Oxford, England) 2010; 26:139–40

7. Anders S, Reyes A, Huber W. Detecting differential usage of exons from RNA-seq data. Genome Research 2012; 22:2008–2017

8. Law CW, Chen Y, Shi W, et al. voom: precision weights unlock linear model analysis tools for RNA-seq read counts. Genome Biology 2014; 15:R29

9. Hartley SW, Mullikin JC. Detection and visualization of differential splicing in RNA-Seq data with JunctionSeq. Nucleic Acids Research 2016; 44:e127

10. Hartley SW, Mullikin JC. QoRTs: a comprehensive toolset for quality control and data processing of RNA-Seq experiments. BMC Bioinformatics 2015; 16:224

11. Hu Y, Huang Y, Du Y, et al. DiffSplice: The genome-wide detection of differential splicing events with RNA-seq. Nucleic Acids Research 2013; 41:

12. Liu R, Loraine AE, Dickerson JA. Comparisons of computational methods for differential alternative splicing detection using RNA-seq in plant systems. BMC Bioinformatics 2014; 15:364

13. Durinck S, Moreau Y, Kasprzyk A, et al. BioMart and Bioconductor: A powerful link between biological databases and microarray data analysis. Bioinformatics 2005; 21:3439–3440

14. Zhu D, Deng N, Bai C. A Generalized dSpliceType Framework to Detect Differential Splicing and Differential Expression Events Using RNA-Seq. IEEE Transactions on Nanobioscience 2015; 14:192–202

15. Quinlan AR, Hall IM. BEDTools: A flexible suite of utilities for comparing genomic features. Bioinformatics 2010; 26:841–842

16. Shen S, Park JW, Lu Z, et al. rMATS: Robust and flexible detection of differential alternative splicing from replicate RNA-Seq data. Proceedings of the National Academy of Sciences 2014; 111:E5593–E5601

17. Alamancos GP, Pagès A, Trincado JL, et al. SUPPA: a super-fast pipeline for alternative splicing analysis from RNA-Seq. bioRxiv 2014; 008763

18. Li B, Dewey CN. RSEM: accurate transcript quantification from RNA-Seq data with or without a reference genome. BMC bioinformatics 2011; 12:323
